# Testicular adrenal rest tumor in infertile man with congenital adrenal hyperplasia: case report and literature review

**DOI:** 10.1590/S1516-31802011000500010

**Published:** 2011-09-01

**Authors:** Giovanni Scala Marchini, Marcello Cocuzza, Rodrigo Pagani, Fábio César Torricelli, Jorge Hallak, Miguel Srougi

**Affiliations:** I Resident, Department of Urology, Hospital das Clínicas (HC), Faculdade de Medicina da Universidade de São Paulo (FMUSP), São Paulo, Brazil.; II MD. Attending physician. Department of Urology, Hospital das Clínicas (HC), Faculdade de Medicina da Universidade de São Paulo (FMUSP), São Paulo, Brazil.; III MD. Professor and Head, Department of Urology, Hospital das Clínicas (HC), Faculdade de Medicina da Universidade de São Paulo (FMUSP), São Paulo, Brazil.

**Keywords:** Adrenal hyperplasia, congenital, Adrenal rest tumor, Infertility, Testis, Microsurgery, Hiperplasia supra-renal congênita, Tumor de resto supra-renal, Infertilidade, Testículo, Microcirurgia

## Abstract

**CONTEXT:**

Synthesis of cortisol and aldosterone is impaired in patients with congenital adrenal hyperplasia (CAH) because of 21-hydroxylase deficiency. Men with CAH have low fertility rates compared with the normal population, and this is related to testicular adrenal rest tumors. Findings of azoospermia in combination with a testicular tumor on ultrasound are likely to have a mechanical cause, especially when in the testicular mediastinum. The preferred treatment method consists of intensive corticoid therapy. However, when the tumor is unresponsive to steroid therapy, surgical treatment should be considered.

**CASE REPORT:**

We present the case of a male patient with CAH due to 21-hydroxylase deficiency who presented a testicular tumor and azoospermia. Treatment with low daily corticoid doses had previously been started by an endocrinologist, but after 12 months, no significant change in sperm count was found. Although the adrenocorticotrophic hormone and 17-hydroxyprogesterone levels returned to normal values, the follicle-stimulating hormone (FSH), luteinizing hormone and testosterone levels remained unchanged. Ultrasound examination confirmed that the testicles were small and heterogenous bilaterally, and revealed a mosaic area at the projection of the testis network bilaterally. Magnetic resonance imaging confirmed the finding. Testicular biopsy revealed the presence of preserved spermatogenesis and spermiogenesis in 20% of the seminiferous tubules in the right testicle. The patient underwent testis-sparing tumor resection. After 12 months of follow-up, there was no tumor recurrence but the patient still presented azoospermia and joined an intracytoplasmic sperm injection program.

## RESUMO

**CONTEXTO:**

Pacientes com hiperplasia adrenal congênita (HAC) por deficiência da 21-hidroxilase podem ter a síntese de cortisol e de aldosterona prejudicada. Homens com HAC têm baixas taxas de fertilidade em comparação com a população normal, e isso está relacionado a tumores testiculares de remanescente adrenal. A associação de azoospermia e tumor testicular sugere uma causa mecânica, principalmente quando o tumor é encontrado no mediastino testicular. O método preferencial de tratamento consiste na corticoterapia intensa. No entanto, quando o tumor não é responsivo à terapia com esteroides, o tratamento cirúrgico deve ser considerado.

**RELATO DE CASO:**

Apresentamos o caso de um paciente do sexo masculino com HAC por deficiência da 21-hidroxilase, portador de tumor testicular e azoospermia. Em consulta prévia com endocrinologista, o paciente começou tratamento com baixas doses diárias de corticoide, porém, após 12 meses de tratamento, não houve mudança significativa no espermograma. Embora os níveis de hormônio adrenocortitrófico e 17-hidroxiprogesterona tenham se normalizado, os níveis séricos de hormônio folículo-estimulante, hormônio luteinizante e testosterona não se alteraram. Exame ultrassonográfico confirmou testículos bilateralmente diminuídos e heterogêneos, além de área em mosaico na projeção da rede testis bilateralmente. Ressonância nuclear magnética confirmou o achado. Biópsia testicular revelou espermatogênese e espermiogênese preservadas em 20% dos túbulos seminíferos no testículo direito. O paciente foi submetido a cirurgia poupadora testicular, com ressecção tumoral. Após 12 meses de acompanhamento, não houve recorrência tumoral, mas o paciente ainda apresentava azoospermia, sendo integrado no programa de injeção intracitoplasmática de espermatozoides.

## INTRODUCTION

Synthesis of cortisol and sometimes of aldosterone is impaired in patients with congenital adrenal hyperplasia (CAH) because of 21-hydroxylase deficiency. Men with CAH have low fertility rates compared with the normal population, and this is related to testicular adrenal rest tumors (TART).^1,2^ Development of the primitive adrenal cortex occurs close to the gonads and TART is considered to be an aberrant adrenal tissue that has descended with the testes.^3^ Pretumor development and growth of these cells is assumed to be adrenocorticotrophic hormone (ACTH) dependent, and undertreatment may play an important role in tumor development. However, intensive glucocorticoid treatment is not always successful in reducing tumor size, and such tumors can be found in as many as 95% of CAH patients.^4-10^ These tumors were first described by Wilkins et al. in 1940 and they are almost always present bilaterally.^11^ Even though they have benign features, they may lead to obstruction of the seminiferous tubules and ultimately infertility, because of their location in the testicular mediastinum.^12-14^ CAH patients not only have anatomical lesions but also have impaired testicular function and hypogonadotropic hypogonadism due to chronic suppression of gonadotropin secretion caused by overproduction of adrenal androgens.^5^ We present the case of a male patient with congenital adrenal hyperplasia due to 21-hydroxylase deficiency who presented a testicular tumor and infertility.

## CASE REPORT

A 39-year-old male patient was referred to our Andrology Division because of primary infertility. He had a past medical history of CAH due to 21-hydroxylase deficiency.

At four years of age, the patient was diagnosed as having the simple virilizing non-salt wasting form of CAH. Treatment with low-dose glucocorticoid (0.5 mg of dexamethasone) was started and he did not need any mineralocorticoid replacement. The patient evolved normally until 22 years of age, when he abandoned his treatment and was lost from follow-up consultations.

He sought medical counseling fifteen years later because of infertility. He was healthy and not taking any medications at that time. He and his wife failed to achieve pregnancy for two years. She did not have any previous offspring, and all possible causes of female infertility were ruled out.

At physical examination, he was seen to have small stature (height: 1.68 m; weight: 78 kg; body mass index: 27.6 kg/m^2^) and small topically softened testicles compatible with testicular atrophy. Semen analyses revealed azoospermia. Serum hormonal screening showed increased levels of follicle-stimulating hormone (FSH) and luteinizing hormone (LH) and low levels of testosterone, albeit within the normal range, thus suggesting testicular failure. The ACTH and 17-hydroxyprogesterone (17-OHP) levels were high, while the levels of other hormones were within the normal range. Treatment with low-dose daily glucocorticoid therapy (0.5 mg of dexamethasone) had been started by an endocrinologist before our first visit, but after twelve months no significant change in sperm count was found. Although the ACTH and 17-OHP levels returned to normal values, the FSH, LH and testosterone levels showed no great improvement (Table 1).

**Table 1:** Hormone levels before and after treatment with glucocorticoid

Serum hormone	Before treatment	After 6 months of dexamethasone	After 12 months of dexamethasone	Normal range
FSH	27 IU/l	34 IU/l	36.1 IU/l	< 10.5 IU/l
LH	30.9 IU/l	18.2 IU/l	27.5 IU/l	1.0-8.4 IU/l
ACTH	827 pg/ml	38 pg/ml	44 pg/ml	< 46 pg/ml
Total testosterone	460 ng/dl	249 ng/dl	374 ng/dl	271-965 ng/dl
Free testosterone	170 pmol/ml	109 pmol/ml	199 pmol/l	131-640 pmol/l
Androstenedione	4.7 ng/ml	1.3 ng/ml	< 0.3 ng/ml	0.3-4.3 ng/ml
17-OHP	122.4 ng/ml	33 ng/ml	2 ng/ml	0.6-3.3 ng/ml
Aldosterone	8.4 ng/dl	16 ng/dl	4.7 ng/dl	1.0-16 ng/dl
Renin activity	2.3 ng/ml/h	17.7 ng/ml/h	4.3 ng/ml/h	1.5-5.7 ng/ml/h
SHBG	46 nmol/ml	65 nmol/ml	55 nmol/l	12-75 nmol/ml

FSH = follicle-stimulating hormone; LH = luteinizing hormone; ACTH = adrenocorticotrophic hormone; 17-OHP = 17-hydroxyprogesterone; SHBG = sex hormone-binding globulin.

**Figure 1:**
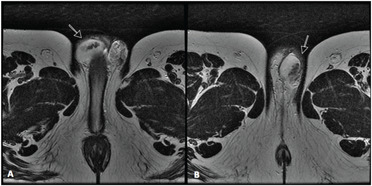
Magnetic resonance imaging (MRI) – Sagittal T2 images revealing low-sign right (A) and left (B) solid elongated serpiginous lesions in the testicular mediastinum (arrows).

**Figure 2:**
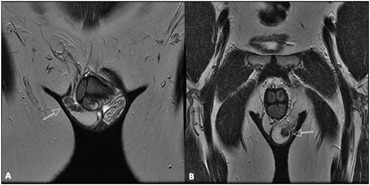
Magnetic resonance imaging (MRI) – Coronal T2 images showing low-sign right (A) and left (B) solid elongated serpiginous lesions in the testicular mediastinum (arrows).

**Figure 3:**
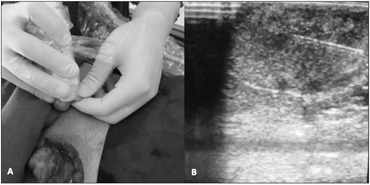
Tumor marking (A) for intraoperative ultrasound examination and needle driving (B).

**Figure 4:**
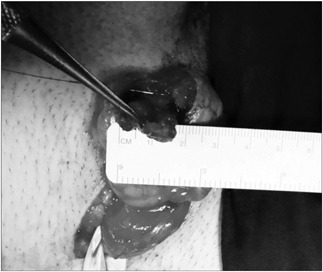
Tumor microdissection and testicular preservation.

Testicular ultrasound examination confirmed that the testicles were small and heterogenous bilaterally (right: 9.1 ml; left: 9.3 ml). It also revealed the presence of a hyperechogenic hypervascularized tumor mosaic area of 1.5 × 1.5 centimeters at the projection of the testis network, in both testicles, thereby suggesting the presence of duct occlusion at this point. Further evaluation using magnetic resonance image (MRI) revealed T2-weighted low-sign bilateral solid elongated serpiginous lesions in the testicular mediastinum, for which adrenal rest tumor was a differential diagnosis (Figures 1 and 2). In order to distinguish testicular failure from obstructive azoospermia, the patient underwent bilateral testicular biopsy. This revealed preserved spermatogenesis and spermiogenesis in 20% of the seminiferous tubules in the right testicle. It also revealed left testicular atrophy characterized by germ cell hypoplasia and basal membrane thickening. The patient underwent testis-sparing tumor resection (Figure3). Bilateral inguinal incisions were made to access the testis and the tumor masses were microdissected using ultrasound guidance (Figure 4). The patient's hospital stay was uneventful. After twelve months of follow-up, ultrasound showed that there was no evidence of tumor recurrence, but the patient still presented azoospermia. He and his wife agreed to join an intracytoplasmic sperm injection (ICSI) program.

## DISCUSSION

Defective conversion of 17-OHP to 11-deoxycortisol, mediated by 21-hydroxylase (CYP21A2), accounts for more than 90% of the cases of CAH.^15-17^ Therapy for CYP21A2 deficiency is directed towards providing glucocorticoid in sufficient doses to reduce the excessive corticotropin-releasing hormone (CRH) levels, corticotropin (ACTH) secretion and hyperandrogenemia.^18-20^ Our patient had the simple virilizing non-salt wasting form of CAH, and after he abandoned his successful glucocorticoid treatment, he presented high serum levels of 17-OHP and infertility.

In 1940, Wilkins et al. reported that testicular tumors occurred in male patients with CAH due to CYP21A2 deficiency, known as so-called testicular adrenal rest tumors (TART).^11^ During the embryological period, steroidogenic cells that are destined to become adrenal and gonadal cells derive from neighboring areas of the coelomic epithelium and are morphologically identical. Their separation takes place in the eighth week of gestation and adrenal cortical tissue may adhere to the gonad and descend with the testis along the course of their supply arteries.^21-26^ In CAH patients, it is believed that these cells can persist and proliferate with preservation of adrenal-like hormone-producing properties. TARTs are ACTH-dependent and may develop during periods of sustained elevation of plasma ACTH levels or regress when glucocorticoid therapy is instituted or intensified.^4,5,27-34^ However, in several studies, no correlation was found between intensive glucocorticoid therapy control and tumor growth.^4,5,8,9,35-37^ Therefore, other factors may interfere. Claahsen-van der Grinten et al. showed that these tumors contain varying amounts of steroid-producing enzymes and ACTH and angiotensin II (AII) receptors, at least at the messenger RNA (mRNA) level, which is believed to have a strong trophic effect on the adrenal gland.^5,38-43^

Depending on the detection method (palpation or ultrasound), the reported prevalence of TART among CAH patients may range from 0 to 95%.^5,27,43^ Absence of palpable tumors does not rule out the existence of TART, and ultrasound is the preferred method for evaluating TART because it is as sensitive as MRI but more accessible.^2^ In our case, MRI was necessary to confirm the ultrasound findings. Histologically, electron microscopy shows that TART resembles Leydig cell tumors, with features that are consistent with steroid-secreting cells. However, unlike Leydig cell tumors, they never contain Reinke crystalloids, are often bilateral and may diminish with corticosteroid therapy.^44^

Different mechanisms have been proposed to explain infertility in males with CAH.^21,22^ Stikkelbroeck et al. demonstrated the presence of testicular dysfunction due to decreased plasma testosterone levels in 35% of the CAH patients that they evaluated, with poor semen quality in 63% of them. This could be caused mechanically by TART, or by local steroid production.^30,45^ At the hypothalamic-pituitary level, secretion of gonadotropins may be suppressed by high levels of adrenal androgens that are aromatized peripherally or in the central nervous system to estrogens.^36,45-48^ In one study, sperm production was found to be impaired in seven of the eleven patients tested, and three (40%) even showed azoospermia. In two of the azoospermic patients, the serum FSH levels were increased, thereby indicating primary testicular dysfunction. In the third azoospermic patient, the serum levels of both LH and FSH were undetectably low, thus suggesting the presence of testicular dysfunction due to hypogonadotropism. With these findings, the authors proposed that routine semen analysis should be performed for such patients. When azoospermia is found in combination with a large testicular tumor on ultrasound, it is likely to have a mechanical cause, especially when in the mediastinum. At this location, large tumors can easily compress the testis network and cause obstructive azoospermia,^49^ as found in our patient. The preferred treatment method for testicular adrenal rest tumors and/or impaired spermatogenesis in patients with CAH is intensive glucocorticoid therapy. This may lead to a decrease in tumor size and improvement of testicular function.^28,47,50-52^ Clomiphene citrate may also be administered, and successful cases have been reported.^46,53^ However, when the tumor is unresponsive to steroid therapy, surgical treatment should be considered, preferably using a testis-sparing procedure.^5^ Recently, Fernandes et al reported a case of a 16-year-old boy who underwent bilateral orchiectomy because of TART that could not be differentiated from a malignant tumor, thus expressing this diagnostic dilemma.^54^

Because of the rarity of TART and its management, we carried out a systematic analysis of the indexed articles published since 1966, in order to provide the best treatment for our patient. We searched using the terms "adrenal rest tumor" and/or "infertility" in the Lilacs (Literatura Latino-Americana e do Caribe em Ciências da Saúde), Embase (Excerpta Medica Database), Medline (Medical Literature Analysis and Retrieval System Online), Scirius and Cochrane Library databases, using DeCS (Descritores em Ciências da Saúde) and MeSH (Medical Subject Headings). Only 10 related references were found in Medline and Scirius, and two in Lilacs (Table 2). Most were descriptive series or case reports. Although proposed by several authors, there is only a single case series addressing surgical management of TART:^2^ Claahsen-van der Grinten et al. evaluated pituitary-gonadal function before and after testis-sparing surgery among CAH patients with TART, and reported that there were no surgical complications and no evidence of residual or recurrent testicular tumor after 22 months. Nonetheless, the semen analysis did not improve after surgery, and persistently low inhibin B levels were found in all patients. The absence of positive effects on testicular function, despite complete removal of the tumors, strongly suggests that irreversible testicular damage preexisted: this was reflected by peritubular fibrosis and tubular hyalinization seen in the testis biopsy specimen that was taken during surgery.^2^ Our patient's semen analyses did not improve with corticosteroid treatment, and the testicular biopsy showed that preserved spermatogenesis and spermiogenesis was present in only 20% of one side. The other testicle was atrophic. No response was obtained after testicular-sparing surgery. It is clear now that, at this stage, surgery can no longer help to restore testicular function. Cryopreservation of the semen or testicular sperm extraction (TESE) with intrauterine injection (IUI) or intracytoplasmic sperm injection (ICSI) can be offered for these patients, as we did in our case, because the fertility prognosis is uncertain.^50^

**Table 2:** Complete literature database search using the terms "adrenal rest tumor" and "infertility" as medical subject headings (MeSH)

Database	Search strategy	Results		
PubMed (Medline)	Adrenal rest tumor (MeSH) AND Infertility	Found: 10	Related: 10	Case reports: 1
				Reviews: 2
				Descriptive series: 7
Embase	Adrenal and rest and tumor	Found: 1	Related: 0	–
Scirius	Adrenal rest tumor (MeSH) AND Infertility	Found: 10	Related: 10	Case reports: 5
				Reviews: 3
				Descriptive series: 2
Lilacs	Adrenal rest tumor (MeSH)	Found: 5	Related: 2	Case reports: 2
Cochrane	Adrenal and rest and tumor	Found: 2	Related: 0	–

Lilacs = Literatura Latino-Americana e do Caribe em Ciências da Saúde; Medline = Medical Literature Analysis and Retrieval System Online; MeSH = Medical Subject Headings.

## CONCLUSION

The prevalence of testicular tumors in male CAH patients is high, despite adequate treatment. Semen production and testosterone secretion may be impaired, especially when large testicular tumors are present. The diagnosis can be achieved using ultrasound or MRI, and early treatment for TART by means of glucocorticoid should be of primary concern. Testis-sparing surgery is feasible, but may not result in testicular function improvement. Alternative fertility techniques and genetic counseling should be offered when all methods fail.
